# Pyoderma Gangrenosum Is Associated With Increased Risk of Inflammatory Pouch-Related Complications: A Retrospective Cohort Study

**DOI:** 10.1093/crocol/otad024

**Published:** 2023-05-14

**Authors:** Ronaldo Paolo Panganiban, Alyssa Tuan, Maxwell Hart, Mathew Pelton, Daniella Mikhail, Sarah Akhtar, Kaleb Bogale, Susan Deiling, Shouhao Zhou, Mathew D Coates, Gregory S Yochum, Walter Koltun

**Affiliations:** Division of Gastroenterology and Hepatology, Department of Internal Medicine, Penn State Hershey Medical Center, Hershey, PA, USA; Division of Colorectal Surgery, Department of Surgery, Penn State Hershey Medical Center, Hershey, PA, USA; College of Medicine, Penn State Hershey Medical Center, Hershey, PA, USA; Department of Medicine, The University of Arizona College of Medicine Tucson, Tucson, AZ, USA; Department of Medicine, Robert Wood Johnson University Hospital, New Brunswick, NJ, USA; Department of Medicine, Robert Wood Johnson University Hospital, New Brunswick, NJ, USA; Department of Internal Medicine, Penn State Hershey Medical Center, Hershey, Pennsylvania; Columbia University Irving Medical Center, New York, NY, USA; Division of Colorectal Surgery, Department of Surgery, Penn State Hershey Medical Center, Hershey, PA, USA; Division of Biostatistics and Bioinformatics, Department of Public Health Science, Penn State Hershey Medical Center, Hershey, PA, USA; Division of Gastroenterology and Hepatology, Department of Internal Medicine, Penn State Hershey Medical Center, Hershey, PA, USA; Division of Colorectal Surgery, Department of Surgery, Penn State Hershey Medical Center, Hershey, PA, USA; Department of Biochemistry and Molecular Biology, Penn State Hershey Medical Center, Hershey, PA, USA; Division of Colorectal Surgery, Department of Surgery, Penn State Hershey Medical Center, Hershey, PA, USA

**Keywords:** pouchitis, pyoderma gangrenosum, IPAA, J-pouch

## Abstract

**Background:**

Pyoderma gangrenosum (PG) is a rare, neutrophilic dermatosis that is a well-established extraintestinal manifestation (EIM) of inflammatory bowel disease. The clinical implications of developing PG in patients with ulcerative colitis (UC) who undergo total proctocolectomy colectomy and ileal pouch anal anastomosis (TPC-IPAA) surgery remain unknown.

**Methods:**

Study participants were selected from patients enrolled in the Carlino Family Inflammatory Bowel and Colorectal Disease Biobank between 1998 and 2021 with a pre-colectomy diagnosis of UC and who underwent TPC-IPAA surgery. A retrospective study comparing patients with PG and those without PG was performed. The outcomes measured included the development of pouchitis, pouchitis classification, presence of pouch fistula, anal fistula, anal stenosis, and pouch failure.

**Results:**

In this study, 357 IPAA patients were included, 10 of whom suffered PG. Patients with PG and without PG had similar demographics and clinical characteristics. Both groups had similar rates of pouchitis (80% in PG patients and 64% in patients without PG, *P* = .504). However, IPAA patients with PG had a higher risk of developing pouch fistula (50% vs 10%, *P* = .002), anal fistula (40% vs 12%, *P* = .031), and Crohn’s-like disease of the pouch (70% vs 15%, *P* = .003) compared to patients without PG. Patients who developed PG prior to their first episode of pouchitis were more likely to eventually experience pouch failure (odds ratio: 20.7, 95% confidence interval: 3.9, 110.7, *q* = 0.003 after false discovery rate adjustment).

**Conclusions:**

Among UC patients who undergo TPC-IPAA surgery, the development of PG portends poor pouch outcomes and is predictive of pouch failure.

## Introduction

Total proctocolectomy with ileal pouch anal anastomosis (TPC-IPAA) is the surgical treatment of choice for patients with medically refractory ulcerative colitis (UC) or UC with dysplasia. The most common complication of this procedure is pouchitis, an inflammatory disorder of the pouch characterized by increased stool frequency, urgency, and abdominal pain. By some estimates, up to 70% of IPAA patients will eventually experience this complication.^1,2^

Pyoderma gangrenosum (PG), a well-known extraintestinal manifestation (EIM) of inflammatory bowel disease (IBD), is a rare and severe inflammatory skin disease characterized by ulceration and necrosis.^3^ An estimated 30% of patients with PG have underlying IBD.^4^ Although PG most commonly affects the lower extremity, it can appear anywhere in the body. Among IBD patients who undergo bowel surgery, the occurrence of peristomal PG (PPG) is a well-recognized phenomenon.^5^

To date, the relationship between pouchitis and PG remains largely unknown. Previous studies have shown that the presence of EIMs either in the pre-colectomy or post-colectomy period increased the risk for pouchitis.^6–11^ Multiple single-patient case reports have documented cases of severe pouchitis with concomitant PG.^12–15^ However, no study has yet associated PG specifically with pouchitis. In this study, our primary aim was to examine the relationship more carefully between PG and pouchitis in a significantly larger cohort of IPAA patients than has previously been studied in this context. We also investigated the correlations between PG and other pouch complications such as fistulizing disease, stricturing disease, Crohn’s-like disease of the pouch (CLDP), and pouch failure.

## Methods

### Study Design and Population

Study participants were selected from patients enrolled in the Carlino Family Inflammatory Bowel and Colorectal Disease Biobank between 1998 and 2021 with a pre-colectomy diagnosis of UC and who underwent TPC-IPAA surgery. A retrospective cohort analysis comparing patients with PG (cases) and those without PG (controls) was performed. Demographic data, baseline characteristics, and clinical outcomes were manually abstracted from the electronic medical record (EMR). Outcomes that were measured include development of pouchitis, pouchitis classification, pouch failure, development of pouch fistula, anal fistula, and anal stenosis. This study was approved by the Penn State College of Medicine Institutional Review Board (IRB Protocol #PRAMSHY98-057). The study design and manuscript were prepared and revised according to the STROBE guidelines.^16^

### Definitions and Classifications

Pouchitis was diagnosed based on the combined assessment of typical clinical symptoms, endoscopy findings, and histology findings as described by the International Ileal Pouch Consortium.^17^ Most patients who developed pouchitis were treated empirically and did not undergo endoscopic evaluation during their first episode of pouchitis. However, over 85% of all patients and over 95% of patients with pouchitis included in the study had eventually undergone at least 1 pouchoscopy. Pouchitis classification (antibiotic-responsive, antibiotic-dependent, antibiotic-refractory) was also defined based on these guidelines.^17^ Crohn’s-like disease of the pouch was defined as antibiotic-refractory pouchitis AND the presence of either fistulizing disease OR stricturing disease. Pouch failure was defined as requiring a permanent diversion ileostomy or pouch excision for the definitive management of pouch complications. Pyoderma gangrenosum was diagnosed based on its typical morphologic appearance and clinical behavior. Tobacco use was assessed in the pre-colectomy and post-colectomy periods. Active smokers in the pre-colectomy period were defined as patients who continued to smoke within 1 month of colectomy, whereas former smokers in the pre-colectomy period were defined as patients who quit more than 1 month prior to colectomy. In the post-colectomy period, active smokers were defined as any smoking after colectomy while former smokers are those who have quit prior to colectomy.

### Statistical Analysis

Statistical analysis was accomplished using the R software.^18^ Fisher’s exact tests and Wilcoxon rank-sum tests were performed where appropriate using the “gtsummary” package.^19^ Penalized logistic regressions with false discovery rate correction for multiple testing were performed using the “mgcv” package.^20^

## Results

### Patient Baseline Characteristics

In this study, a total of 357 patients with UC who underwent IPAA surgery were included. Of these patients, 3% (10 out of 357) had a diagnosis of concomitant PG. The demographics and clinical characteristics of the study patients are summarized in Table 1. No statistically significant differences in sex, race, tobacco use, or IBD family history between these groups were noted. In addition, both groups had similar UC-specific characteristics, including age at UC diagnosis, indications for colectomy, time between UC diagnosis and colectomy, pre-colectomy biologic exposure, age at colectomy, surgical approach to pouch creation, comorbid primary sclerosing cholangitis (PSC), and length of follow-up since IPAA construction.

**Table 1.** Patient demographics and clinical characteristics.

Characteristic	*N*	Overall, *N* = 357^a^	No PG*, N* = 347^a^	PG present, *N* = 10^a^	*P* value^b^
Sex = male	357	215 (60)	211 (61)	4 (40)	.205
Race	357				>.999
White, not Hispanic		325 (91)	315 (91)	10 (100)	
White, Hispanic		12 (3)	12 (4)	0 (0)	
Black, not Hispanic		7 (2)	7 (2)	0 (0)	
Other		13 (4)	13 (4)	0 (0)	
Age at UC diagnosis, years	351	27.0 (20.3, 36.9)	26.5 (20.2, 37.0)	31.3 (26.0, 33.3)	.257
Pre-colectomy biologic treatment	345	126 (37)	123 (37)	3 (30)	.800
Medically refractory		288 (88)	280 (88)	8 (100)	
Dysplasia		38 (12)	38 (12)	0 (0)	
Time between UC diagnosis and colectomy, years	346	5.2 (2.0, 11.8)	5.8 (2.0, 12.0)	2.7 (0.8, 3.9)	.163
Age at colectomy	352	35.6 (27.0, 45.8)	35.5 (27.0, 45.7)	35.9 (31.8, 46.2)	.795
Surgical approach	352				.173
1-stage		28 (8)	28 (8)	0 (0)	
2-stage		145 (41)	143 (42)	2 (20)	
Modified 2-stage		60 (17)	56 (16)	4 (40)	
3-stage		119 (34)	115 (34)	4 (40)	
Pre-colectomy tobacco use	348				>.999
Active		34 (10)	33 (10)	1 (10)	
Former		96 (28)	93 (27)	3 (30)	
Never		218 (63)	212 (63)	6 (60)	
Post-colectomy tobacco use	353				>.999
Active		38 (11)	37 (11)	1 (10)	
Former		102 (29)	99 (29)	3 (30)	
Never		213 (60)	207 (60)	6 (60)	
IBD family history	352	88 (25)	87 (25)	1 (10)	.462
PSC	357	38 (11)	38 (11)	0 (0)	.608
Follow-up since IPAA, y	357	10.7 (4.4, 18.3)	10.9 (4.4, 18.4)	7.4 (3.5, 15.4)	.423

Abbreviations: IBD, inflammatory bowel disease; IPAA, ileal pouch anal anastomosis; IQR, interquartile range; PG, pyoderma gangrenosum; UC, ulcerative colitis.

^a^ *n* (%); Median (IQR).

^b^Fisher’s exact test; Wilcoxon rank-sum test.

### Pouchitis and Pouchitis-Related Complications

Patients who developed PG had a similar pouchitis rate to the patients who did not develop PG (80% vs 64%, *P* = .504; Table 2). The difference between the relative rates of anal stricturing disease between these cohorts was also not statistically significant (31% vs 50%, *P* = .296). However, patients with PG were more likely to suffer from pouch fistulae (50% vs 10%, *P* = .002) and anal fistulae (40% vs 12%, *P* = .031). Overall, PG patients were also more likely to be diagnosed with CLDP (70% vs 15%, *P* = .002) and suffer pouch failure (70% vs 5%, *P* < .001).

**Table 2.** Pouchitis-related complications of IPAA surgery.

Characteristic	*N*	Overall, *N* = 357^a^	No PG, *N* = 347^a^	PG present, *N* = 10^a^	*P* value^b^
Pouchitis	357	231 (64)	223 (64)	8 (80)	.504
Pouchitis classification	357				.003
No pouchitis		126 (35)	124 (36)	2 (20)	
Antibiotic-responsive		106 (30)	105 (30)	1 (10)	
Antibiotic-dependent		46 (13)	46 (13)	0 (0)	
Antibiotic-refractory		20 (6)	20 (6)	0 (0)	
CLDP		59 (17)	52 (15)	7 (70)	
Pouch fistula	356	39 (11)	34 (10)	5 (50)	.002
Anal stricture	356	111 (31)	106 (31)	5 (50)	.296
Anal fistula	357	47 (13)	43 (12)	4 (40)	.031
Pouch failure	357	23 (6)	16 (5)	7 (70)	<.001

Abbreviations: CLDP, Crohn’s-like disease of the pouch; IQR, interquartile range; PG, pyoderma gangrenosum.

^a^ *n* (%); median (IQR).

^b^Fisher’s exact test; Wilcoxon rank-sum test.

### PG-Specific Characteristics

Of the 10 patients who developed PG, 6 were diagnosed with this skin condition prior to their first episode of pouchitis (Table 3, patients A, B, C, D, E, and J). Among these 6 patients, 5 were diagnosed with PPG during their multistage pouch construction procedure (patients A, B, C, D, and E). Patients A, C, D, and E developed PG after their total colectomy procedures. Patient C had recurrent PG after the construction of her IPAA with diverting loop ileostomy, but this resolved with stomal closure. Patients A, B, and E eventually suffered pouch failure with patient E experiencing recurrent PG around his permanent diversion ileostomy. Lastly, patient J had lower extremity PG that was diagnosed more than 7 years prior to his colectomy.

**Table 3.** Clinical characteristics of individual PG patients.

Patient	Age at TPC (years)	Sex	Pouchitis classification	Surgical technique	PG site	PG context	PG treatment	PG resolution	Pouch failure
A	47	Female	CLDP	Modified 2-stage	Peristomal	After colectomy	Prednisone, cyclosporine	Resolved	Failed, diverted
B	49	Male	CLDP	3-stage	Peristomal	After IPAA	NA	Resolved	Failed, diverted
C	31	Female	No pouchitis	3-stage	Peristomal	After colectomy, recurred after IPAA with diversion	1^st^ PG: Prednisone, cyclosporine 2^nd^ PG: infliximab, stoma closure	Resolved	Intact
D	36	Female	No pouchitis	Modified 2-stage	Peristomal	After colectomy	Prednisone, Stoma closure	Resolved	Intact
E	36	Male	CLDP	Modified 2-stage	Peristomal	After colectomy, recurred after permanent diversion for management of medically refractory CLDP	1^st^ PG: cyclosporine, prednisone, stoma closure recurrent PG: dapsone, intralesional triamcinolone, prednisone, adalimumab, infliximab, ustekinumab	Not Resolved	Failed, diverted
F	23	Female	CLDP	3-stage	Peristomal	After diversion ileostomy for medically refractory CLDP, subsequently underwent pouch excision	Intralesional triamcinolone, adalimumab	Resolved	Failed, excised
G	44	Female	CLDP	2-stage	Peristomal	After diversion ileostomy for medically refractory CLDP	Prednisone, intralesional triamcinolone	Resolved	Failed, diverted
H	25	Female	CLDP	Modified 2-stage	Peristomal, Lower extremity	Peristomal PG after pouch excision, unclear when lower extremity PG initially diagnosed	Intralesional triamcinolone, dapsone	NA	Failed, excised
I	33	Male	CLDP	3-stage	Peristomal	After diversion ileostomy for medically refractory CLDP, subsequently underwent pouch excision	Intralesional triamcinolone	Resolved	Failed, excised
J	50	Male	Antibiotic-responsive	2-stage	Lower extremity	Before colectomy	Infliximab, adalimumab, ustekinumab	Not Resolved	Intact

Abbreviations: CLDP, Crohn’s-like disease of the pouch; IPAA, ileal pouch anal anastomosis; IQR, interquartile range; PG, pyoderma gangrenosum; TPC, total proctocolectomy.

On the other hand, the remaining 4 patients with PPG developed PG as part of the surgical management of their medically refractory pouchitis (patients F, G, H, and I). All 4 of these patients experienced pouch failure. Patients F, G, and I developed PG after the creation of diverting loop ileostomy for the management of recalcitrant pouchitis. Patients F and I eventually underwent pouch excision as well. Patient H developed PPG after pouch excision, but she also suffered from recurrent lower extremity PG of unclear duration.

Resolution of PG was documented in 7 of the 10 patients. There were 2 patients whose PG failed to resolve despite various interventions including multiple biologic therapies (patients E and J). Of note, both patients continue to follow-up with our institution at the time of manuscript writing. The single patient (patient H) who had both PPG and lower extremity PG was lost to follow-up without documentation of PG resolution.

### PG as Predictor of Pouch Complications

To assess whether PG predicts pouch complications, a subanalysis that only included patients who developed PG prior to their first episode of pouchitis (*n* = 6) was performed. Again, no significant differences in baseline clinical characteristics and demographics were observed (Table S1). In this population, the occurrence of PG remained significantly associated with pouch failure (50% vs 5%, *P* = .002; Table S2). However, we did not observe a significant association between PG and other outcome variables, such as pouchitis, pouchitis classification, pouch fistula, anal stricture, and anal fistula. Given these findings, we performed the univariable logistic regression analyses using pouch failure as the dependent variable (Table 4). The presence of PG, tobacco use pre- and post-colectomy, age at colectomy, and presence of PSC were included as predictors. Patients with missing values for any of these predictors were excluded. In total, 339 patients were included in this subanalysis, 18 of whom suffered pouch failure. Among the included variables, the significant predictor of pouch failure was the presence of PG (odds ratio [OR]: 20.7, 95% confidence interval [CI]: 3.9, 110.7, *q* = 0.003) and Post-Colectomy Tobacco Use (Never Smoker vs Active user, OR: 0.2, 95% CI: 0.1, 0.6, *q* = 0.020). Similar results were found for the presence of PG (OR: 34.6, 95% CI: 5.7, 208.1, *q* = 0.001) in the multivariable logistic regression analysis although the protective effect of never smoking loses statistical significance (Table S3).

**Table 4.** Univariable penalized logistic regression to evaluate predictors of pouch failure among patients who develop PG prior to pouchitis.

Characteristic	*N*	Event *N*	OR	95% CI	*P* value	*Q* value^a^
Presence of PG	339	18	20.7	3.9, 110.7	<.001	0.003
Pre-colectomy tobacco use	339	18				
Active			—			
Former			0.7	0.2, 2.8	.582	0.582
Never			0.5	0.1, 1.9	.296	0.414
Post-colectomy tobacco use	339	18				
Active			—			
Former			0.3	0.1, 0.9	.039	0.090
Never			0.2	0.1, 0.6	.006	0.020
Age at colectomy	339	18	1.0	0.9, 1.0	.170	0.298
PSC	339	18	0.4	0.1, 3.4	.438	0.511

Abbreviations: Cl, confidence interval; OR, odds ratio; PG, pyoderma gangrenosum; PSC, primary sclerosing cholangitis.

^a^False discovery rate correction for multiple testing.

## Discussion

In this study, we explored the association of PG with pouchitis and pouchitis-related complications. Although the development of PG among UC patients who undergo IPAA surgery does not result in a significantly higher rate of pouchitis, a diagnosis of concomitant PG in this population portends poor pouch outcomes. Specifically, we report that the diagnosis of PG is associated with a higher likelihood of developing pouch fistulae and anal fistulae. Additionally, in this cohort, the occurrence of PG is associated with the development of CLDP. Ultimately, we demonstrate that PG is a harbinger of pouch failure.

Patients with IBD typically develop PG in their lower extremities.^21^ By contrast, among our UC patients who underwent IPAA surgery, 8 out of the 10 PG cases occurred in the peristomal region and 1 patient had both PPG and lower extremity PG. More than half of our PPG cases were first diagnosed during part of their multistage pouch construction procedure. A reasonable approach to management of PPG was suggested by O’Brien et al.^22^ Stoma closure when possible has been suggested to be the most successful therapy for these patients.^5,23^ Most of our PPG cases were treated with a combination of topical and systemic medical therapy. More recently, successful treatment of PG with the use of anti-TNF antibodies, combined anti-IL12/23 antibodies, and JAK/STAT inhibitors have been reported in small randomized control trials and single case reports.^24^ Larger studies are needed to establish the efficacy of these biologics and small molecules in the treatment of PG. It also remains to be seen whether previous exposure to these medications affects their efficacy in treating PG.

In our subanalysis, we limited our PG cohort to those who developed PG prior to their diagnosis of pouchitis. This was performed to evaluate PG as a predictor of pouch outcomes. Even though this reduced our PG cohort by 4 patients, we were still able to show that PG is predictive of pouch failure. Interestingly, one of the 6 PG patients included in this analysis (patient J) was immediately reinstated on biologic therapy after his colectomy. This was done as part of his lower extremity PG treatment. Although it cannot be conclusively proven, it is possible that this continuous biologic therapy prevented him from developing CLDP. Despite this, he still experienced occasional episodes of antibiotic-responsive pouchitis. On the other hand, in this subanalysis, we were not able to show statistically significant associations between PG and other pouch complications. The incongruence between these 2 observations is most likely due to a reduced statistical power stemming from a smaller PG cohort.

In addition, our univariable logistic regression showed a statistically significant protective effect of never smoking in preventing pouch failure. By our definition outlined above, post-colectomy never smokers are lifetime nonsmokers. This finding agrees with our previously published data on this group of patients suggesting that former smokers and active smokers were more likely to develop pouchitis compared to nonsmokers.^25^ The statistical significance of the protective effect of never smoking in preventing pouch failure is, however, lost in our multivariable logistic regression. This asks for further validation considering the small number of pouch failures in our dataset.

Nevertheless, considering our above findings, patients who develop PG prior to or at the time of pouch construction should be counseled about their potential increased risk for severe pouchitis phenotypes. Conceivably, patients who experienced PPG and eventually suffered from pouchitis may benefit from a more rapid escalation of medical therapy to prevent fistulizing disease. Rather than maximizing antibiotic therapy, a more expedient transition to immunomodulators, small-molecule inhibitors, or biologic therapy could reasonably be considered for these patients. Future studies are needed to determine if these suggestions will be backed by evidence-based medicine.

The remaining cases of PPG occurred in the context of the surgical management of severe pouchitis. In this clinical scenario, the occurrence of pouchitis and diagnosis of CLDP preceded the development of PPG. For this set of patients, PPG failed to be a predictor of disease as it emerged after CLDP had already been diagnosed. Whether PG develops prior to or after pouchitis, the development of PG among UC patients who undergo TPC-IPAA surgery should prompt IBD providers to ensure close patient follow-up given the association of PG with poor pouch outcomes. It might be prudent for IBD providers to implement a low threshold for endoscopic and histologic assessment of the pouch in this population. Judicious use of NSAIDs should also be emphasized.^2,26^

Our study has several strengths. Whereas previous studies have shown that EIMs are associated with pouchitis; to our knowledge, our report is the first retrospective study that specifically investigated the relationship between PG and inflammatory pouch complications. In this study, each patient profile was manually inspected in the EMR, and data from available scanned paper records were also reviewed. Additionally, we were able to apply a rigorous definition of pouchitis in which more than 95% of patients with pouchitis eventually underwent endoscopic evaluation. While there is no standard definition for CLDP, we applied a stringent criterion for this disease entity which required both antibiotic-refractoriness AND Crohn’s-like behavior (stricturing or fistulizing disease) to be present. Finally, our study is one of the largest of its kind to date and has a long period of evaluation where the overall median follow-up time is more than a decade.

There are, however, some limitations to our study as well. PG is rare—the 2.8% prevalence of PG within our cohort matches the generally accepted 1%–5% prevalence of PG among IBD patients.^27^ Even though we were able to find statistically significant associations between PG and pouch complications, we could not fully assess its utility as a *predictor* of pouchitis and other pouch outcomes because a significant number of our patients had PG *after* their diagnosis of pouchitis and CLDP. The size of our PG cohort also did not allow for the identification of which PG patients will experience treatment-resistant PG nor did it permit us to conclude if pouch excision results in PG resolution. Further, our study was performed in a tertiary care center where the majority of our patients are non-Hispanic Caucasians, which raises the question of the external validity. Finally, despite our best efforts, other potentially informative clinical data (presence of backwash ileitis at the time of colectomy, erythrocyte sedimentation rate, C-reactive protein, fecal calprotectin, etc.) could not be included due to a high degree of missingness.

In conclusion, in this large retrospective study with over a decade of median patient follow-up, we demonstrate that while the occurrence of PG in patients who undergo TPC-IPAA surgery is not associated with increased risk of pouchitis, it is associated with the development of pouch fistula, anal fistula, and CLDP. Importantly, the appearance of PG is predictive of pouch failure in UC patients with IPAA. Our findings allow IBD providers to assess the risk of poor pouch outcomes more accurately in this patient population.

## Supplementary Material

otad024_suppl_Supplementary_Material

## Data Availability

The data underlying this article cannot be shared publicly due to confidential protected health information. The data will be shared on reasonable request to the corresponding author.
