# Supplementary material for: Pyoderma Gangrenosum Is Associated With Increased Risk of Inflammatory Pouch-Related Complications: A Retrospective Cohort Study
Source: Crohns Colitis 360. 2023 May 14;5(3):otad024. doi: 10.1093/crocol/otad024 (PMC10474334; doi:10.1093/crocol/otad024)
Supplement: otad024_suppl_Supplementary_Material [file otad024_suppl_supplementary_material.docx]

# **Supplemental Table 1.** Patient demographics and clinical characteristics. Only patients who develop PG prior to their first episode of pouchitis were included for analysis.

| Characteristic | N | Overall, N = 357^1^ | No PG, N = 347^1^ | PG present, N = 6^1^ | *P* value^2^ |
| --- | --- | --- | --- | --- | --- |
| Sex = Male | 353 | 214 (60) | 211 (61) | 3 (50) | 0.684 |
| Race | 353 |  |  |  | >0.999 |
| White, Not Hispanic |  | 321 (91) | 315 (91) | 6 (100) |  |
| White, Hispanic |  | 12 (3) | 12 (4) | 0 (0) |  |
| Black, Not Hispanic |  | 7 (2) | 7 (2) | 0 (0) |  |
| Other |  | 13 (4) | 13 (4) | 0 (0) |  |
| Age at UC diagnosis, y | 347 | 27.0 (20.2, 36.9) | 26.5 (20.2, 37.0) | 31.3 (28.8, 32.8) | 0.246 |
| Colectomy indication | 323 |  |  |  | >0.999 |
| Medically refractory |  | 285 (88) | 280 (88) | 5 (100) |  |
| Dysplasia |  | 38 (12) | 38 (12) | 0 (0) |  |
| Time between UC diagnosis and colectomy, y | 342 | 5.6 (2.0, 12.0) | 5.8 (2.0, 12.0) | 3.7 (2.9, 15.8) | 0.856 |
| Age at colectomy | 348 | 35.7 (27.1, 46.0) | 35.5 (27.0, 45.7) | 41.5 (35.8, 48.3) | 0.243 |
| Surgical approach | 348 |  |  |  | 0.225 |
| 1-Stage |  | 28 (8) | 28 (8) | 0 (0) |  |
| 2 Stage |  | 144 (41) | 143 (42) | 1 (17) |  |
| Modified 2-Stage |  | 59 (17) | 56 (16) | 2 (33) |  |
| 3-Stage |  | 117 (34) | 115 (34) | 3 (50) |  |
| Pre-colectomy Tobacco Use | 344 |  |  |  | 0.426 |
| Active |  | 33 (10) | 33 (10) | 0 (0) |  |
| Former |  | 96 (28) | 93 (27) | 3 (50) |  |
| Never |  | 215 (62) | 212 (63) | 3 (50) |  |
| Post-colectomy Tobacco Use | 349 |  |  |  | 0.449 |
| Active |  | 37 (11) | 37 (11) | 0 (0) |  |
| Former |  | 102 (29) | 99 (29) | 3 (50) |  |
| Never |  | 210 (60) | 207 (60) | 3 (50) |  |
| IBD Family History | 348 | 87 (25) | 87 (25) | 0 (0) | 0.343 |
| PSC | 353 | 38 (11) | 38 (11) | 0 (0) | 0.999 |
| Follow up since IPAA, y | 353 | 10.8 (4.4, 18.3) | 10.9 (4.4, 18.4) | 6.5 (1.9, 15.4) | 0.411 |

^1^n (%); Median (IQR)
^2^Fisher’s exact test; Wilcoxon rank sum test

# **Supplemental Table 2.** Pouchitis-related complications of IPAA-surgery assessed among patients who developed PG prior to their first episode of pouchitis.

| Characteristic | N | Overall,  N = 353^1^ | No PG,  N = 347^1^ | PG present,  N = 6^1^ | *P* value^2^ |
| --- | --- | --- | --- | --- | --- |
| Pouchitis | 353 | 227 (64) | 223 (64) | 4 (67) | >0.999 |
| Pouchitis Classification | 353 |  |  |  | 0.291 |
| No Pouchitis |  | 126 (36) | 124 (36) | 2 (33) |  |
| Antibiotic-responsive |  | 106 (30) | 105 (30) | 1 (17) |  |
| Antibiotic-dependent |  | 46 (13) | 46 (13) | 0 (0) |  |
| Antibiotic-refractory |  | 20 (6) | 20 (6) | 0 (0) |  |
| CLDP |  | 55 (15) | 52 (15) | 3 (50) |  |
| Pouch fistula | 352 | 35 (10) | 34 (10) | 1 (17) | 0.469 |
| Anal stricture | 352 | 109 (31) | 106 (31) | 3 (50) | 0.379 |
| Anal fistula | 353 | 45 (13) | 43 (12) | 2 (33) | 0.171 |
| Pouch failure | 353 | 19 (5) | 16 (5) | 3 (50) | 0.002 |

^1^n (%); Median (IQR)
^2^Fisher’s exact test; Wilcoxon rank sum test

**Supplemental Table 3.** Multivariable penalized logistic regression to evaluate predictors of pouch failure among patients who develop PG prior to pouchitis.

| Characteristic | N | Event N | OR^1^ | 95% CI^1^ | *P* value | *Q* value^2^ |
| --- | --- | --- | --- | --- | --- | --- |
| Presence of PG | 339 | 18 | 34.6 | 5.7, 208.1 | <0.001 | 0.001 |
| Pre-Colectomy Tobacco Use | 339 | 18 |  |  |  |  |
| Active |  |  | -- |  |  |  |
| Former |  |  | 4.6 | 0.4, 57.0 | 0.232 | 0.325 |
| Never |  |  | 1.4 | 0.1, 20.4 | 0.791 | 0.791 |
| Post-Colectomy Tobacco Use | 339 | 18 |  |  |  |  |
| Active |  |  | -- |  |  |  |
| Former |  |  | 0.1 | 0.0, 1.0 | 0.053 | 0.185 |
| Never |  |  | 0.2 | 0.0, 1.8 | 0.140 | 0.244 |
| Age at colectomy | 339 | 18 | 1.0 | 0.9, 1.0 | 0.098 | 0.228 |
| PSC | 339 | 18 | 0.7 | 0.1, 6.1 | 0.773 | 0.791 |

^1^OR = Odds Ratio, Cl = Confidence Interval

^2^False discovery rate correction for multiple testing
